# Putting ICAP to the test: how technology-enhanced learning activities are related to cognitive and affective-motivational learning outcomes in higher education

**DOI:** 10.1038/s41598-024-66069-y

**Published:** 2024-07-15

**Authors:** Christina Wekerle, Martin Daumiller, Stefan Janke, Oliver Dickhäuser, Markus Dresel, Ingo Kollar

**Affiliations:** 1 University of Augsburg, Universitätsstr. 10, 86135 Augsburg, Germany; 2 University of Mannheim, A 5, 6, 68159 Mannheim, Germany

**Keywords:** Human behaviour, Statistics

## Abstract

Digital technology is considered to have great potential to promote learning in higher education. In line with the Interactive, Constructive, Active, Passive (ICAP) framework, this seems to be particularly true when instructors stimulate high-quality learning activities such as constructive and interactive learning activities instead of active and passive learning activities. Against the background of a lack of empirical studies in authentic, technology-enhanced instructional settings, we investigated the cognitive and affective-motivational effects of these learning activity modes in technology-enhanced higher education courses. To this end, we used 3.820 student assessments regarding 170 course sessions for which the teachers stated the learning activities students were engaged in. Results of multilevel structural equation modelling highlight the importance of technology-enhanced interactive learning activities for students’ perception of learning and the potential negative consequences of passive learning activities for affective-motivational outcomes. However, the superiority of constructive and interactive learning activities compared to passive and active learning activities for cognitive and affective-motivational outcomes was not supported by the findings. Instead, the findings point to potential differential effects of the individual learning activities within one activity mode. Future research should follow up on these effects to gain a more fine-grained understanding of how technology-enhanced learning activities can be optimized to enhance students’ learning outcomes.

## Introduction

Over 50 years of research have led to the conclusion that the general impact of digital technology on students’ performance in higher education is characterized by a small positive effect at best^1,2^. Yet, considering more specific relationships, a meta-analysis documented a positive medium effect of digital games on students’ learning achievement in STEM education when compared to traditional instruction^3^. In contrast, a meta-analysis on the effects of teaching with PowerPoint found no differences in students’ learning outcomes when comparing PowerPoint-based teaching with traditional chalkboard-based teaching^4^. Taken together, these and other meta-analyses^5–7^ indicate that it is not so much technology per se that determines its effects, but rather *how it is used* by teachers and students. Accordingly, researchers in the past and present have recommended moving away from so-called media comparisons. Instead, it should be investigated how to best instructionally implement different technologies in classrooms by focusing on the quality of the technology-enhanced learning activities^8,9^.

To this end, scholars have already proffered suitable conceptual frameworks. Specifically, a prominent instructional model that assumes qualitative differences between four different modes of observable learning activities (interactive, constructive, active, and passive) in terms of students’ associated learning is the ICAP framework^10–13^. Despite the ICAP framework becoming more and more established in the field of educational research, there is still a lack of empirical studies that investigate the effects of different learning activity modes on cognitive outcomes, particularly in authentic technology-enhanced higher education settings. Also, numerous theories point to the fact that learning is not just a cognitive, but also an affective-motivational endeavour. However, this aspect has not yet been considered in ICAP research. Against this backdrop, in the present study, we investigated the effects of different technology-enhanced learning activity modes on students’ perceptions of their learning, situational interest and joy in higher education.

### Quality of technology implementation based on the ICAP framework

The ICAP framework^10–13^ differentiates four observable modes of learning activities that are assumed to be associated with different knowledge change processes, resulting in different cognitive learning outcomes. These four modes of passive, active, constructive, and interactive learning will be outlined in the following:

Learning activities are defined as *passive* if learners attend towards the learning material and absorb its information without any other observable learning activity. Passive activities might include listening to a podcast or watching a video and are believed to typically only allow isolated storage of information in terms of knowledge-change processes. As a result, learners might only recall the acquired knowledge in identical contexts or when receiving cues for activation. Note that common classroom activities in higher education, such as plenary discussions and presentations, are considered predominantly passive because only a few students will be constructive or interactive at a particular time and most students would only follow the discussion and presentation without doing anything else^13^.

*Active* learning includes learning activities in which learners observably and physically manipulate learning materials, including rotating virtual three-dimensional objects, pausing or rewinding online videos, and underlining (important) text passages of a hypermedia text. The active activity mode is considered to encourage the activation of prior knowledge and linking new information and prior knowledge by adapting and complementing schemata. These knowledge construction processes are supposed to facilitate an application of the acquired knowledge in contexts similar to the acquisition context.

Student learning activities are identified as being *constructive* if students create learning products that go beyond the information that were originally provided by the learning materials. Examples for constructive activities are creating a digital concept map or visualization. A cognitive process that is thought to be typically connected to constructive activities is inferring new knowledge from the activated prior knowledge and the linked knowledge by means of revising, replacing, or restructuring stored knowledge and identifying lacking knowledge. Consequently, a transfer of knowledge should be enabled.

Finally, *interactive* learning activities take place when students engage in conversations with one or more learning partners characterized by students’ statements being of a constructive nature (instead of passive or active) and a sufficient degree of turn taking. This is supposed to occur when students argue for or against a position in a discussion forum or when students jointly develop a concept for an explanatory video using a visualization tool. Interactive learning activities are assumed to facilitate the acquisition of new knowledge by linking knowledge with the help of learning material (see constructive learning activities) and contributions of the learning partners, in a co-inferring process. In terms of learning outcomes, a joint construction of new (innovative) knowledge should be likely, meaning that students generate knowledge they would not have been able to generate on their own.

Integrating the assumptions on the four learning activity modes, the ICAP hypothesis is as follows: engaging in interactive learning activities should be superior to engaging in constructive learning activities which in turn should be superior to engaging in active learning activities regarding students’ knowledge acquisition. Engaging in passive learning activities is meant to be associated with the lowest outcomes. In addition, Chi and colleagues^11^ consider the gulf between *shallow activities* that do not include the process of inferring (meaning passive and active learning activities), and *deep activities* that do include the process of inferring (meaning constructive and interactive learning activities) to be bigger than within the shallow and deep learning activities. This perspective aligns well with other theoretical models (such as Select-Organize-Integrate^14^) which differentiate between non-generative and generative learning activities^15^. As the ICAP hypothesis is supposed to be “applicable to all students, all grade levels, and for all content domains”^16^ when considering certain prerequisites and limitations^11,13^, it can provide higher education teachers with (potentially) observable indicators for high-quality technology-enhanced teaching.

### Research on ICAP in (technology-enhanced) higher education settings

To test the ICAP hypothesis, first, Chi^10^ and Chi and Wylie^13^ presented a reanalysis of around 40 existing studies (not exclusively regarding higher education). Despite seemingly supporting the ICAP hypothesis, this analysis may be criticized due to a positive selection resulting from a non-systematic search and presentation of findings. This circumstance is reflected in the lack of information on the search procedure and is emphasized by Chi and Wylie themselves^13^. Second, an experimental study^18^ was performed in which the four learning activity modes were manipulated in a controlled setting. After an individual pretest and reading of an introductory text, all participants were randomly assigned to one of the ICAP conditions, received a fixed time frame to work on the allocated learning activity, and finally took an individual posttest. The results largely confirmed the ICAP hypothesis. However, in less controlled or more authentic learning contexts in which the ICAP hypothesis has been tested so far, the results are more mixed^17–20^. Yet, in most of these studies, at least the assumed effects of deep and shallow learning activity modes could be found. For example, students’ constructive/interactive contributions in a MOOC online discussion forum were found to be more conducive to students’ knowledge gain than active contributions^20^. In a study on the effects of peer instruction (discussion of answers in small groups after answering quiz questions with audience response systems) with a passive, constructive, and interactive condition, only students learning interactively substantially surpassed those who were learning passively^21^. While these study results are generally in line with the ICAP hypothesis, or at least do not point to a different hierarchy of learning activities, there are several important aspects that previous studies have not addressed yet: first, most studies have not considered authentic technology-enhanced learning contexts. In such contexts students are typically not only asked to perform a single activity to approach a topic (e.g., watching a video) like it is often the case in experimental studies. Instead, they are expected to combine multiple learning activities, move from one to another or switch between them (e.g., watching a video, answering quiz questions, discussing answers with peers, writing a collaborative blog entry on the video). If such contexts were addressed, students’ self-report data on their technology-enhanced learning activities were used only on a coarse-grained level (assessment on technology-enhanced learning activities in all the course at the end of the term^21^).

Second, few studies have considered multiple learning activities (e.g., listening, reading, watching) within one learning activity mode (e.g., passive) and investigated whether these have comparable effects on knowledge acquisition, as implied by the ICAP model. Though Chi and Wylie^13^ acknowledge that some learning activities might be more cognitively challenging than others within one mode, the ICAP framework does not make any assumptions about their differential effectiveness.

Third, despite their established importance for students’ learning processes^22^, students’ motivation (e.g., situational interest) and emotions (e.g., joy) have not been included in ICAP-based studies to date. Regarding the enhancement of students’ motivation as well as positive emotions, theories point to the relevance of learning arrangements in which students experience autonomy and control over a task, consider the task at hand useful and have a sense of belonging^23–27^. We believe that these processes are more likely to occur when students engage in constructive and interactive learning activities compared to passive and active learning activities. This might be due to the kind of tasks that ask students to generate new, not solely teacher-provided knowledge and therefore engage in (joint) deep processing of the learning content. Empirical findings in support of these assumptions originate from meta-analyses on problem-based learning and computer-supported collaborative learning. In both fields, small to medium positive effects on students’ motivational outcomes could be observed when compared to more traditional and mostly non-collaborative control conditions^28–31^. They might be well interpreted based on the ICAP learning modes, as problem-based learning mostly focuses on constructive and interactive learning activities^32^ and computer-supported collaborative learning mostly focuses on interactive learning activities. Thus, in ICAP terms, this might mean that deep interactive and constructive learning activities should be more conducive to students’ affective-motivational outcomes than shallow passive and active learning activities.

### The present research

Based on the ICAP framework and the findings presented above, we investigated the effects of the four different technology-enhanced learning activity modes reported by teachers on students’ cognitive as well as affective-motivational outcomes. We asked higher education teachers of different domains to report on students’ learning activities that occurred in their actual classes while asking students to report on their learning outcomes in end-of-session evaluations. By assessing various course sessions, we aimed to capture authentic learning settings in which a wide range of learning activities might occur over time. We assumed that constructive and interactive learning activities (i.e., “deep” learning activities in Chi and Wylie’s terms) are more positively related to students’ learning (cognitive outcome, H1) and students’ situational interest and joy (affective-motivational learning outcomes, H2) than passive and active (i.e., “shallow”) learning activities. Furthermore, we sought to explore to what degree different effects of single learning activities within one mode on the cognitive and affective-motivational learning outcomes might occur.

## Methods

To investigate our hypotheses, we asked 87 university teachers to provide details on the individual learning activities their students engaged in a particular session. Simultaneously, the students evaluated their learning in these sessions using end-of-session paper-and-pencil surveys (students’ perceptions of learning, situational interest, joy). We used the data from all sessions that were responded to by the teachers and evaluated by at least five students. This data is part of a larger project on faculty motivation where we investigated different research questions to the present work (focused on faculty members and their motivations, instead of technology-enhanced learning activities). Specifically, in research questions unrelated to the present investigation, it has been studied to which end faculty motivations are associated with faculty members’ professional learning^33^, how stable and context specific faculty members’ goals and emotions are^34^, and how they relate to their basic need satisfaction^35^ (reported in each session by the faculty members, alongside the assessment of their learning activities). This data was also used in another publication in which these faculty motivations and emotions were compared to analogous assessments during COVID-19^36^. Further, in past research on this data, we investigated how students’ assessments of teaching quality are linked to faculty members’ teaching motivations^37^. This last publication contains partial data overlap with the present investigation in that the two of the same dependent variables were considered (students’ evaluations of learning and joy), but with a subsample only. The study was approved by the University of Mannheim IRB board, carried out in accordance with the relevant guidelines and regulations, and participation was voluntary and incentivized through small thank-you gifts provided to the participating teachers. Through e-mails to the department heads, we informed about the study and asked faculty members to participate. Those who agreed to participate received further information to be sent to their students, informing them about the study. Prior to participation, all participants provided informed consent.

### Sample

The sample stems from a medium-sized university in the south of Germany, the University of Augsburg. Altogether, our sample consisted of 3.820 student assessments regarding 170 sessions of 42 different courses. The participating students had a mean study duration of 3.5 semesters (*SD* = 2.1), 81% of them were women. The teachers were on average 42.1 (*SD* = 11.6) years old, 59.6% of them held a PhD, and about 2 out of 3 were female (68.4%). They were recruited from multiple departments and came from the fields of education sciences, sociology, sports sciences, mathematics, economics, and psychology. At the time of data collection, the University of Augsburg had seven faculties, which are located in different buildings. Most of the included students and teachers were part of the faculty of philosophy and social sciences. We primarily recruited teachers from this faculty due to practical considerations (the study involved research assistants administering the surveys in each session).

### Measures

For each session, the teachers made assessments regarding students’ learning activities using a half-structured and open-ended scale (see Supplementary Material S1/2 online). Therein, we first asked the teachers about the total length of the session. This was followed by an instruction in which we detailed what we meant by “types of learning activities” that students performed during the respective sessions (e.g., listen, take notes, summarize content, develop ideas with other students). Likewise, we then illustrated what we meant by “digital technology tools” that the teachers might have used to facilitate these learning activities (presentation software, quiz tools, text editors, video editing software, etc.). Participants were then asked to chronologically list the different learning activities students engaged in during their session, how long these were and whether digital tools were used (e.g., generation of digital mind maps), addressed (e.g., discussion about a shown video), or not used/addressed (e.g., reading of printed text) for each of them. The development of the scale was based on previous studies^21^^,^^38^ in which teachers’ (intended) ICAP-based use of technology was measured. In the present research, we specifically assessed time spent on the individual learning activities to allow for a differentiated measurement of each mentioned activity. Subsequently, using a coding scheme based on Chi and Wylie^13^ and Chi et al.^11^ two trained undergraduate research assistants performed a content analysis. The independent coders manually rated the mentioned activities along the ICAP dimensions and classified each mentioned activity as either passive (e.g., watch video), active (e.g., search of information), constructive (e.g., concept mapping), or interactive (e.g., developing group presentation). Data which could not be identified as an activity due to lacking information was classified as other (e.g., “computer”). Following typical recommendations^39^, we calculated inter-rater reliability based on a random subsample of 10% of the data, which yielded a good reliability of Cohen’s *k* = 0.88. For the analyses, we then used the duration of each of the four activity modes relative to the overall duration of the session (e.g., if the session was 90 min long, and a total of 45 min were reported for digital, passive learning activities, then the score was 45/90 = 0.5, reflecting that half of the learning time in that session was spent on these respective activities).

The sessions were evaluated by the students regarding their perceptions of learning, situational interest, and joy. For *students' perceptions of learning*, we used two items of the respective subscale from the SEEQ^40–42^ that assessed students’ evaluation in terms of learning (e.g., “I have learned a lot in today’s session”; Spearman-Brown: *r* = 0.71). *Situational interest* was assessed with a single item^43^ that read as “I found today’s session very interesting”. Finally, *joy* was assessed with the following single item^44^: “In today’s session I felt joy”. All items were responded to on a Likert-type scale ranging from 1 (*do not agree at all*) to 8 (*agree completely*). Intraclass correlations (student assessments per each individual session: ICC1 = 0.07–0.12; ICC2 = 65–0.75) pointed to substantial shared portions of variance in students’ session-specific assessments that were reliably assessed among them.

### Analyses

We conducted multilevel analyses (student evaluations, nested in different course sessions) in which we regressed students’ perceptions of learning, situational interest, and joy on the learning activity modes reported by the teachers regarding the different sessions. Simultaneously, we controlled for teachers’ age and gender (given documented differences based on these aspects regarding student evaluations of teaching quality and teachers’ perceptions and use of digital technology^45^). The multilevel model and its variables are depicted in the Supplementary Material S3 online. Note that time spent on the four different learning activity modes was not linearly dependent, as teachers also spent time on other activities. We used the model constraint command to identify which regression weights were statistically significantly different from each other. The analyses were conducted in Mplus (Version 8.1) using MLR as an estimator.

## Results

Descriptively, we found that teachers reported on average three (*M* = 2.95, *SD* = 1.29) different learning activities that students engaged in their sessions. Almost all of them could adequately be classified into one of the four ICAP learning activity modes, only 5% of the mentioned learning activities were not classifiable and placed into the “other” category. Typically, this was due to the teachers not having described the respective learning activity in sufficient detail.

Most of the time, these learning activities were facilitated with digital technologies: 70% of learning time was spent with direct use of digital technology (e.g., generation of digital mind maps), 9% while addressing digital technology (e.g., discussion about a shown video), and 19% without use of digital technology (e.g., reading of printed text). In the following, we focus on the learning activities facilitated with direct use of digital technology. We found that for an average lesson with a time of 80 min, passive learning activities had the highest share with 41.5 (*SD* = 44.6) minutes, followed by active learning activities with 12.5 (*SD* = 25.4) minutes, constructive learning activities with 3.3 (*SD* = 10.5) minutes, and interactive learning activities with 4.5 (*SD* = 11.7) minutes. Passive learning activities (252 in total) primarily included listening (66), discussion (48), and (student) presentations (41); active learning activities (64 in total) included taking notes (15), textual work (5), and using work sheets (5), constructive activities (32 in total) included summarizing information (6), developing ideas (6), and making a video (4), interactive learning activities (39 in total) primarily consisted of group work (29), but also partner discussions (3) and preparation of group presentations (3).

### Associations between teachers’ use of ICAP learning modes with digital technology and students’ learning outcomes (hypothesis 1 and 2)

Results of the multilevel modelling are reported in Table 1. Regarding hypothesis 1, the findings showed that students reported statistically significantly better learning, the more teachers reported to have used interactive digital learning activities in their sessions. Conversely, and regarding hypothesis 2, the more teachers reported using passive learning activities in a session, the less joy reported students to have experienced in the respective session. All other regression weights were not statistically significant. The effect of passive learning time on joy was statistically smaller than the effect of constructive learning time on joy (*p* < 0.05), all other regression weight comparisons did not reach statistical significance.

**Table 1:** Results of multilevel modelling associations between use of ICAP learning modes and students’ perceptions of outcomes.

	Learning			Interest			Joy		
	β	*SE*	*p*	β	*SE*	*p*	β	*SE*	*p*
Model 1: associations between use of ICAP learning modes and students learning outcomes									
Passive	0.05	0.09	0.55	0.03	0.08	0.69	**−0.15**	**0.07**	** < 0.05**
Active	−0.04	0.12	0.74	−0.05	0.12	0.66	0.04	0.10	0.38
Constructive	−0.01	0.09	0.88	−0.06	0.09	0.50	0.06	0.09	0.51
Interactive	**0.11**	**0.05**	** < 0.05**	0.01	0.08	0.98	−0.01	0.05	0.17
Model 2: exploratory analyses on differential relationships for different passive learning activities									
Listening	−0.04	0.09	0.60	−0.05	0.08	0.55	**−0.18**	**0.07**	** < 0.05**
Plenary discussion	−0.08	0.06	0.22	−0.08	0.07	0.23	−0.06	0.05	0.18
Presentation	0.08	0.07	0.25	**0.10**	**0.05**	** < 0.05**	−0.01	0.05	0.81

*N* = 3820 student assessments nested in 170 course sessions that teachers reported on regarding the employed learning activities. Both models yielded a good fit to the data (CFI > 0.99, TLI > 0.99, RMSEA < 0.02, SRMR < 0.03).

Statistically significant values are in bold.

### Exploratory analyses on differences between different passive learning activities

In the second model, we followed up on the passive learning activities that were largely reported by the faculty members. We regressed the same dependent variables on the three most prevalent types of passive learning activities: listening, plenary discussion, and (student) presentation. The results showed that students reported higher situational interest, the longer learning with presentations was reported by the teachers. Further, students reported experiencing less joy, the longer teachers reported to evoke passive listening activities. The other effects were not statistically significant. Comparisons of the regression weights confirmed that the effects of presentations on students’ perceptions of learning and situational interest were statistically larger than those of listening and plenary discussions on their perceptions of learning and situational interest.

## Discussion

To evaluate higher education teachers’ technology implementation not only from a perspective of quantity but also quality, ICAP holds the potential to serve as a valuable framework. However, several questions regarding its application in authentic (technology-enhanced) learning contexts remain open. With the present research, we tried to remedy some of these issues by investigating to what degree the ICAP hypothesis holds true for cognitive as well as affective-motivational outcomes, in authentic technology-enhanced higher education course sessions, and to what degree there might be differences in the effects of single technology-enhanced learning activities within one particular learning activity mode.

Descriptively, the associations between the four learning activity modes, students' perceptions of learning, situational interest as well as joy were roughly in line with the ICAP hypothesis^13^, findings on problem-based learning and computer-supported collaborative learning^28–32^. However, only technology-enhanced interactive learning activities could explain students’ more positive perceptions of their learning, whereas engagement in the other learning activity modes did not seem to be substantially aligned with students’ perceptions of learning. Regarding affective-motivational outcomes, only passive learning activities were negatively associated with joy. We could only observe a significant difference between the shallow passive and the deep constructive learning activity mode for joy in favour of the constructive activity mode. Consequently, the pattern of results provides empirical support for the “end points” of the ICAP-continuum, i.e., passive and interactive learning activity modes, and at the very least does not contradict the generally assumed hierarchy of learning activity modes. Yet, we could not confirm our hypotheses. We likely have to consider different factors to explain the lacking effects.

First, the present study differs from previous ICAP-based studies in that it was implemented in authentic learning settings. In such settings, different learning activities are typically combined or arranged in sequences. Therefore, the execution of a certain learning activity might enhance or impair the execution of a subsequent activity. For example, in two studies students showed better cognitive skills when receiving a modelling example (passive instruction) before problem-solving (constructive instruction) in an alternate manner compared to when being confronted with problem-solving tasks prior to modelling examples, particularly when students had low prior knowledge^46^. Thus, in line with Roscoe et al.^47^, we suggest further investigations on the sequence of learning activities to determine if activity order, e.g., from low to high engagement or vice versa, influences students' efforts and learning outcomes.

Second, teachers might tend to report students’ learning activities according to their intended student behaviours instead of the kind of learning they actually encourage by their instruction or their perceived student behaviours. The following observation confirms this explanation: Despite their initial intentions (e.g., designing a constructive or interactive lesson), teachers were inclined to use directives and provide questions in activity sheets that might initiate active learning activities (e.g., label, match^11^). Consequently, even though teachers’ assessment of students’ activities might be more accurate than students’ own assessment^21^, teachers’ bias should be still accounted for in future research (by introducing ICAP-oriented lesson transcript-based assessments of lesson tasks^48^).

Third, the exploratory analysis of the passive learning activities revealed that single learning activities within one mode can produce different effects. In concrete terms, while (student) presentations produced positive effects on situational interest, listening (to teachers) produced negative effects on students’ joy. In addition, presentations were more conducive to students’ perceptions of learning and situational interest than listening and plenary discussion. These differences within one mode might have resulted in the lacking effects of the learning activities by cancelling each other's positive and negative effects out. Consequently, the results raise the question if the ICAP learning activity modes might be too coarse-grained to capture learning activities and their effects accurately.

Despite these study-specific explanations, the theoretical assumptions stated in the ICAP framework might itself impose limitations on the study of learning activities. Though there might be an association between directly observable behaviour and cognitive processes, it is rather a matter of probability than a deterministic relation^13^. However, elaboration and other deep-level learning processes are mainly responsible for students’ learning gains.

Irrespective of the strengths of our study (including the authentic learning contexts, the pairing of student and teacher reports, and the fine differentiation of learning activities), methodological aspects also must be considered that might have influenced our findings: First, the teachers in our sample tended to only make generic notes on students’ learning activities which could have resulted in a too inclusive or exclusive coding and lacking differences between the activities. Second, students’ perceptions of learning as measured in our study focused on the quantity of learning (e.g., “I have learned a lot in today’s session”). In other words, we might not have been able to capture the quality of learning in terms of deep knowledge-change processes assumed to take place when engaging in deep learning activities. Third, and related, the study only relies on self-reported (albeit stemming from both teachers and students) data which might have resulted in an assessment bias. Fourth, our sample contained data regarding courses from multiple subjects, primarily from the humanities and social sciences. This could limit generalizability to other fields (e.g., medical education), and there might be subject-specific effects that could be informative for future research to follow up on. Last, due to the low frequency of reported single learning activities within the active, constructive and interactive learning activity mode, we could only compare single learning activities within one mode but not across different modes.

In conclusion, the ICAP framework is considered helpful as a theoretical model to describe and classify technology-enhanced learning activities in terms of the quality of technology implementation. It might assist higher education teachers to plan, implement, and evaluate their technology-enhanced teaching. Our results particularly establish the importance of technology-enhanced interactive learning activities for students’ learning success as well as the potential negative consequences of technology-enhanced passive learning activities for students’ joy. Thus, higher education teachers should be supported in using technology in their teaching in a more interactive and less passive way. However, regarding future empirical research, further details in the ICAP framework might need to be amended. Based on our results, we suggest following up on the effects of combining or sequencing different learning activities and of single learning activities within one mode.

### Supplementary Information

Supplementary Information.

## Data Availability

The dataset and the analytical code underlying the presented analyses are available from the corresponding author on reasonable request. We will publish the full dataset after an embargo period.
