# Supplementary material for: Putting ICAP to the test: how technology-enhanced learning activities are related to cognitive and affective-motivational learning outcomes in higher education
Source: Sci Rep. 2024 Jul 15;14:16295. doi: 10.1038/s41598-024-66069-y (PMC11250844; doi:10.1038/s41598-024-66069-y)

# Supplementary Materials

S1: Scale to assess students learning activities (Translated to English)

**Today's session lasted approximately ____ minutes.**

In the following, we would like you to tell us (1) what learning activities (e.g. listening, taking notes, summarising content, developing ideas with other students) you think the students did in today's session, (2) to what extent digital media (e.g. presentation software, quiz tools, text editors, video editing software) were used, thematised or not used/thematised in this and (3) roughly how long each activity lasted.

|  | **During this learning activity, digital media were … by the teacher/student.** | | | **Time** |
| --- | --- | --- | --- | --- |
| Please briefly name the students' learning activities in this column (chronologically; maximum six activities) | …used. (e.g. creating a digital mind map) | …thematised. (e.g. discuss online video shown) | … not used or thematised. (e.g. reading printed text) | in Min. |
| 1. | o | o | o | ____ |
| 2. | o | o | o | ____ |
| 3. | o | o | o | ____ |
| 4. | o | o | o | ____ |
| 5. | o | o | o | ____ |
| 6. | o | o | o | ____ |

*Note*: Please make only one cross per line for media support.

S2: Scale to assess students learning activities (Original, German Version)

**Die heutige Sitzung hat ca. ____ Minuten gedauert.**

Im Folgenden möchten wir gerne von Ihnen wissen, (1) welche **Lernaktivitäten** (z.B. Zuhören, Notizen machen, Inhalte zusammenfassen, mit anderen Studierenden Ideen entwickeln) die Studierenden Ihrer Meinung nach in der heutigen Sitzung durchgeführt haben, (2) inwiefern hierbei **digitale Medien** (z.B. Präsentationssoftware, Quiztools, Texteditoren, Videobearbeitungssoftware) genutzt, thematisiert oder nicht genutzt/thematisiert wurden und (3) **wie lange** die Aktivitäten jeweils grob gedauert haben.

|  | **Während dieser Lernaktivität wurden digitale Medien durch die Lehrperson/Studierenden…** | | | **Zeit** |
| --- | --- | --- | --- | --- |
| Bitte benennen Sie in dieser Spalte kurz die Lernaktivitäten der Studierenden (chronologisch; maximal sechs Aktivitäten) | …genutzt. (z.B. Erstellen digitaler Mindmap) | …thematisiert. (z.B. Diskutieren über gezeigtes Onlinevideo) | …nicht genutzt/ thematisiert. (z.B. Lesen von ausgedrucktem Text) | in Min. |
| 1. | o | o | o | ____ |
| 2. | o | o | o | ____ |
| 3. | o | o | o | ____ |
| 4. | o | o | o | ____ |
| 5. | o | o | o | ____ |
| 6. | o | o | o | ____ |

*Hinweis: Bitte machen Sie Sie bei Medienunterstützung jeweils nur ein Kreuz pro Zeile.*

S3: Depiction of the multilevel model with its variables


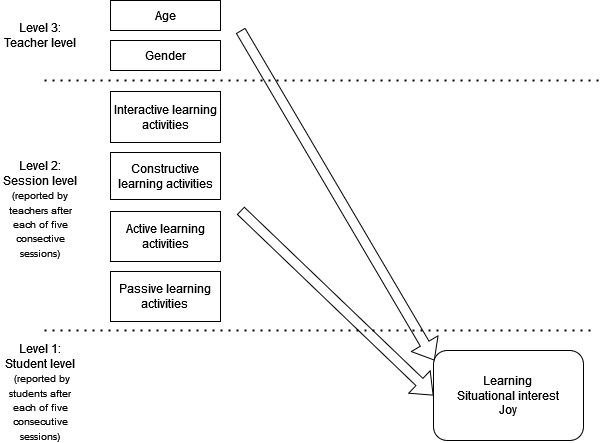

Supplement: Supplementary file 1 — Supplementary Information. [file 41598_2024_66069_MOESM1_ESM.docx]
